# Nuclear KIT induces a NFKBIB-RELA-KIT autoregulatory loop in imatinib-resistant gastrointestinal stromal tumors

**DOI:** 10.1038/s41388-019-0900-9

**Published:** 2019-07-30

**Authors:** Yuan-Shuo Hsueh, Hui Hua Chang, Yan-Shen Shan, H. Sunny Sun, Jonathan Alfred Fletcher, Chien-Feng Li, Li-Tzong Chen

**Affiliations:** 1 National Institute of Cancer Research, National Health Research Institutes, Tainan, Taiwan; 2 International Center for Wound Repair and Regeneration, National Cheng Kung University, Tainan, Taiwan; 3 Institute of Clinical Pharmacy and Pharmaceutical Sciences, College of Medicine, National Cheng Kung University, Tainan, Taiwan; 4 School of Pharmacy, College of Medicine, National Cheng Kung University, Tainan, Taiwan; 5 Department of Pharmacy, National Cheng Kung University Hospital, College of Medicine, National Cheng Kung University, Tainan, Taiwan; 6 Department of Pharmacy, National Cheng Kung University Hospital, Dou-Liou Branch, Yunlin, Taiwan; 7 Institute of Clinical Medicine, College of Medicine, National Cheng Kung University, Tainan, Taiwan; 8 Department of Surgery, National Cheng Kung University Hospital, Tainan, Taiwan; 9 Institute of Molecular Medicine, College of Medicine, National Cheng Kung University, Tainan, Taiwan; 10 Bioinformatics Center, National Cheng Kung University, Tainan, Taiwan; 11 Department of Pathology, Brigham and Women’s Hospital and Harvard Medical School, Boston, MA USA; 12 Department of Pathology, Chi-Mei Foundation Medical Center, Tainan, Taiwan; 13 Department of Biotechnology, Southern Taiwan University of Science and Technology, Tainan, Taiwan; 14 Department of Internal Medicine, National Cheng Kung University Hospital, National Cheng Kung University, Tainan, Taiwan; 15 Department of Internal Medicine, Kaohsiung Medical University Hospital, Kaohsiung Medical University, Kaohsiung, Taiwan

**Keywords:** Oncogenes, Target identification, Drug development

## Abstract

Gastrointestinal stromal tumors (GISTs) are frequently driven by auto-activated, mutant KIT and have durable response to KIT tyrosine kinase inhibitor. However, acquired resistance is an increasing clinical issue in GIST patients receiving front-line imatinib therapy. Our previous studies showed the colocalization of KIT with DAPI-stained nuclei in GIST cells without knowing the role of nuclear KIT in GIST tumorigenesis. In this article, we first identified the binding of nuclear KIT to the promoter of *NFKB inhibitor beta (NFKBIB)* by chromatin immunoprecipitation (ChIP) sequencing and ChIP assays, which was accompanied with enhanced NFKBIB protein expression in GIST cells. Clinically, high NCCN risk GISTs had significantly higher mean expression levels of nuclear phospho-KIT and NFKBIB as compared with those of intermediate or low/very low-risk GISTs. Conversely, downregulation of NFKBIB by siRNA led to RELA nuclear translocation that could bind to the *KIT* promoter region and subsequently reduced KIT transcription/expression and the viability of GIST cells. These findings were further confirmed by either RELA overexpression or NFKB/RELA inducer, valproic acid, treatment to result in reduced KIT expression and relative cell viability of imatinib-resistant GIST cells. Combining valproic acid with imatinib showed significantly better growth inhibitory effects on imatinib-resistant GIST48 and GIST430 cells in vitro, and in the GIST430 animal xenograft model. Taken together, these results demonstrate the existence of a nuclear KIT-driven NFKBIB-RELA-KIT autoregulatory loop in GIST tumorigenesis, which are potential targets for developing combination therapy to overcome imatinib-resistant of KIT-expressing GISTs.

## Introduction

Gastrointestinal stromal tumors (GISTs) are the most common type of mesenchymal neoplasm of the gastrointestinal tract [1, 2]. Mutated and autophosphorylated KIT has been identified as a therapeutic target for ~80% of GISTs. Primary mutations most commonly occur in the juxta-membrane domain (exon 11), and occasionally occur in the extracellular domain (exon 9), the ATP-binding domain (exon 13/14), and the activation loop domain (exon 17). Pharmacological inhibition of KIT phosphorylation by the tyrosine kinase inhibitor (TKI) imatinib (IM, Gleevec®) is the first-line treatment for GISTs. However, 50% of GIST patients experience disease progression within 2 years of IM treatment [3]. The well-known mechanisms underlying the development of acquired IM resistance include the acquisition of secondary mutations in exon 13, 14, or 17 of KIT or the expression of wild-type (WT) KIT [4]. Although the TKIs sunitinib (Sutent®) and regorafenib (Stivarga®) are the second- and third-line drugs, respectively, TKI resistance is a critical issue [5]. In GISTs, mutated KIT undergoes autophosphorylation and constitutively activates downstream signaling pathways [6, 7]. However, the mechanism underlying increased KIT expression and KIT-related tumorigenesis in GISTs has not been fully explored. Accordingly, further investigations into the mechanisms of KIT-driven tumorigenesis and the identification of new therapeutic targets are needed to develop treatment for patients with TKI-resistant, KIT-expressing GISTs.

Increasing evidence has shown that receptor tyrosine kinases, such as epidermal growth factor receptor (EGFR) and insulin-like growth factor 1 receptor (IGF1R), can translocate into the nucleus and mediate gene expression, resulting in tumorigenesis and drug resistance. Lin et al. first defined nuclear EGFR as a transcriptional cofactor that could bind to the *CCND1* promoter and upregulate *CCND1* expression [8]. Clinically, elevated nuclear EGFR expression is an indicator of poor treatment outcomes in cancer patients. Similarly, IGF1R is another membrane receptor that can translocate into the nucleus, bind to putative enhancer sites in gDNA, and drive gene expression [9]. In addition, Warsito et al. and Sarfstein et al. identified a positive regulatory loop involving nuclear IGF1R-mediated LEF1/TCF-derived gene expression, which, in turn, modulates *IGF1R* gene expression [10, 11].

In our previous studies, we found that KIT colocalized with DAPI-stained nuclei in IM-resistant, mutant KIT-expressing GIST48 and GIST430 cells [12, 13]. However, it is unknown whether KIT can locate in the nucleus. In addition, the role of nuclear KIT in GIST tumorigenesis has not been fully elucidated. In this study, we aimed to investigate the role of nuclear KIT in IM-resistant GIST48 and GIST430 cells. Using chromatin immunoprecipitation sequencing (ChIP-seq) and ChIP assays, we found that nuclear KIT could bind to the *NFKB inhibitor beta (NFKBIB)* promoter region and regulate NFKBIB expression. Moreover, we investigated the roles of NFKBIB and its active component, NFKB, in relative cell viability and KIT regulation in GIST cells. We also demonstrated that targeting NFKBIB and NFKB with valproic acid (VPA, Depakine®) alone or in combination with IM achieved a better inhibitory effect on tumor growth in IM-resistant GISTs in vitro and in vivo. Our results help elucidate the role of nuclear KIT and provide potential therapeutic targets for IM-resistant, KIT-expressing GISTs.

## Results

### KIT localizes to the cytoplasm and nucleus in IM-resistant GIST cells

**Fig. 1:**
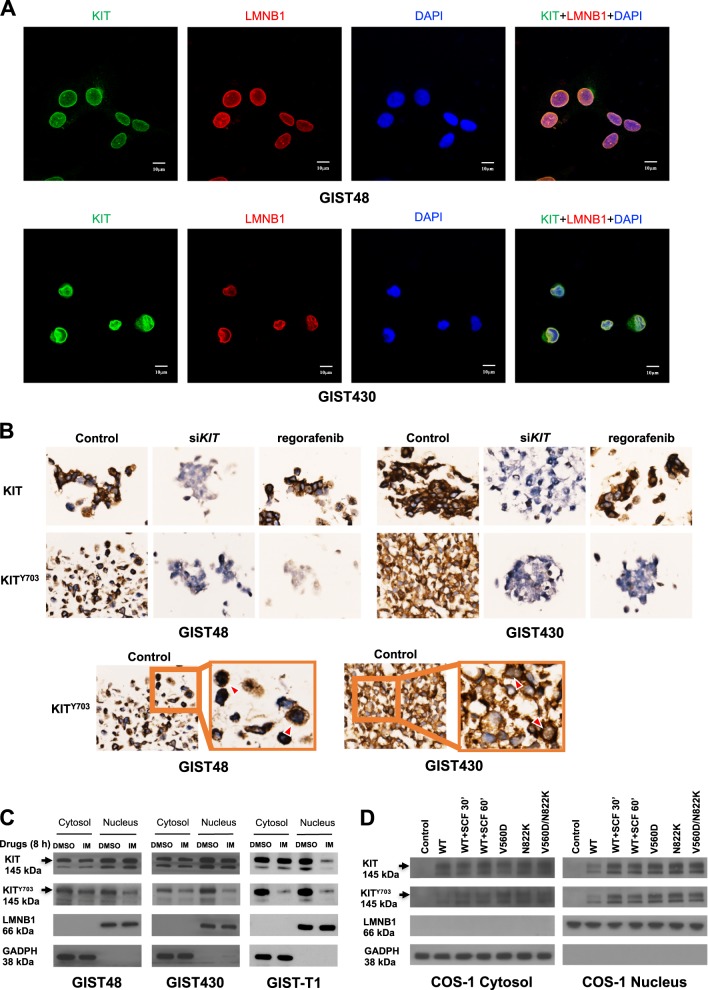
Distribution of KIT in the cytoplasm and nucleus of GIST cells. **a** GIST48 and GIST430 cells were stained using antibodies against KIT and LMNB1. After the cells were immunostained, they were visualized by confocal microscopy, and images were acquired through the Cy2, rhodamine, and DAPI channels (×1000). The data were derived from representative images of five fields/picture for each sample. **b** Cells were transfected with 150 nM siRNA targeting *KIT* for 72 h or treated with 1 μM regorafenib for 8 h. The cell blocks were analyzed by immunohistochemistry staining against phosphorylated KIT (KIT^Y703^) and total KIT. GIST cells were treated with 1 μM IM for 8 h (**c**), and COS-1 cells were transfected with various KIT mutants and treated with or without SCF for indicated time (**d**). The cells were fractionated to separate the cytoplasmic and nuclear proteins and analyzed by immunoblotting for KIT^Y703^ and total KIT. LMNB1 and GAPDH were used as nuclear and cytoplasmic markers, respectively. All experiments were repeated at least three times

Our previous data showed that KIT colocalized with DAPI-stained nuclei in IM-resistant GIST cells [12, 13]. To confirm such observation, we examined the distribution of KIT in GIST48 and GIST430, the two IM-resistant cell lines whose secondary *KIT* mutation in exon 17^D820A^ and exon 13^V654A^, respectively, are responsible for acquired resistance in >50% of IM-resistant cases of GIST. Immunofluorescence staining showed the colocalization of KIT with the nuclear envelope marker LMNB1 and with the DAPI-stained nuclei in both GIST cell lines (Fig. 1a). The z-stack series of images were shown in Fig. S1. The antibody specificity of phospho-KIT (KIT^Y703^) was validated in cell blocks treated with a KIT inhibitor regorafenib or a siRNA targeting *KIT* (Fig. 1b). In those cells treated with si*KIT*, the immunoexpression of both KIT and KIT^Y703^ were downregulated in both cell lines; while in those treated by regorafenib, only KIT^Y703^ was downregulated. These results confirmed the antibody specificities on immunohistochemistry. In addition to GIST48 and GIST430 cells, an IM-sensitive cell line with primary *KIT* exon 13^K642E^ mutation, GIST-T1, was analyzed. Protein fractions from all three GIST cell lines showed that KIT was expressed in both the cytoplasm and the nucleus (Fig. 1c). After IM treatment, both cytoplasmic and nuclear KIT^Y703^ were apparently inhibited in IM-sensitive GIST-T1 cells, but were only partially inhibited in IM-resistant GIST48 and GIST430 cells. Furthermore, KIT with mutations in exon 11^V560D^, exon 17^N822K^, and exon 11^V560D^/17^N822K^, representing IM-sensitive, partially responsive, and IM-resistant mutations, respectively, were autophosphorylated and overexpressed in the cytoplasm and the nucleus in KIT^null^ COS-1 cells (Fig. 1d). Interestingly, the phosphorylation levels of wild-type (WT) KIT induced by its ligand stem cell factor (SCF) for 30 and 60 min were correlated with the KIT expression levels in the nucleus. These results also indicated the antibody specificities on immunoblotting. Taken together, these results indicated the expression of phosphorylated KIT in the nucleus of GIST cells could be modulated by TKI as their cytoplasmic counterpart did.

### Nuclear KIT binds to the *NFKBIB* promoter region and regulates NFKBIB expression

**Fig. 2:**
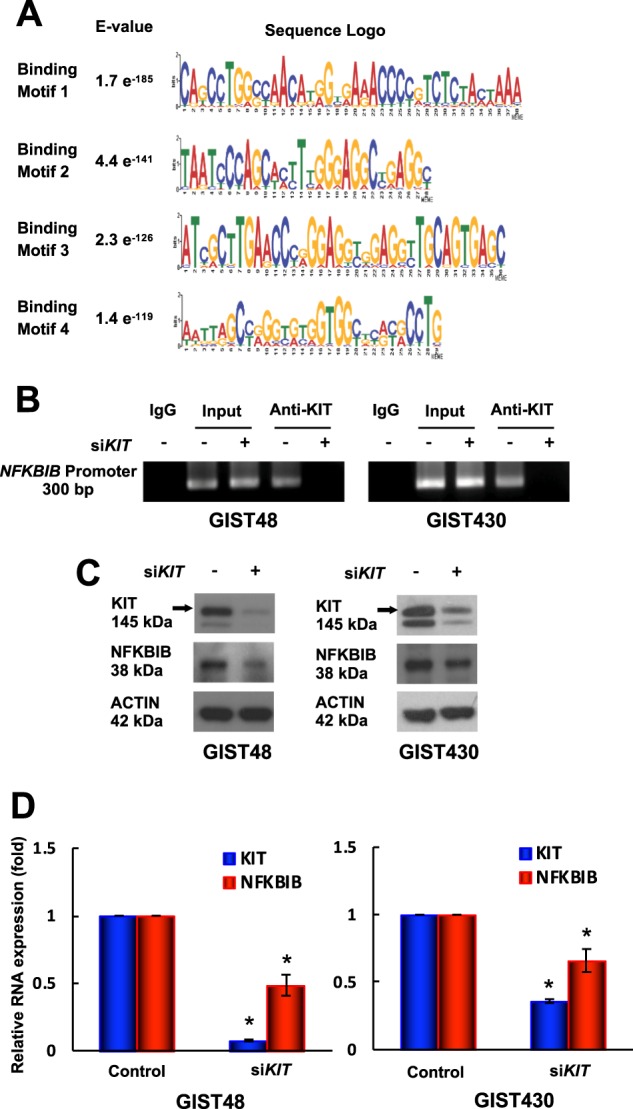
Role of nuclear KIT in GIST48 and GIST430 cells. Chromatin obtained from GIST48 cells was cross-linked, sheared, immunoprecipitated using an anti-KIT antibody, and analyzed using next-generation sequencing (NGS). Short reads obtained from NGS were mapped to the reference genome. Enriched reads (compared with a normal IgG-immunoprecipitated control) were adjusted using filters, and their distribution in the genome was determined. **a** Logos were obtained by running MEME-ChIP with 300-bp summits of the top 600 KIT-bound specific ChIP-seq peaks. The numbers next to the logos indicated the occurrence of the motifs and the statistical significance (E-value). **b**–**d** GIST48 and GIST430 cells were transfected with 150 nM siRNA targeting *KIT* for 72 h. **b** The chromatin was cross-linked, sheared, immunoprecipitated using an anti-KIT antibody, and amplified by PCR. Chromatin that was sheared but not immunoprecipitated was used as an input control. Protein (**c**) and RNA (**d**) extracted from parental and *KIT*-silenced cells were analyzed by immunoblotting and real-time PCR, respectively. Actin served as an internal control for both protein and RNA loading. All experiments were repeated at least three times. The data are expressed as the means ± SD of three or more independent experiments. *p < 0.05

To further explore the biologic functions of nuclear KIT in GIST cells, we extracted nuclear proteins from GIST48 cells, immunprecipitated nuclear KIT-bound DNA, and analyzed them by ChIP-seq. The data showed that KIT could bind to the promoter regions of 442 genes (Fig. S2A). Further bioinformatics analyses of the experimental and control groups identified four KIT-binding motifs with significant E-values (<e^−100^) compared with that of the shuffled background (>e^006^) (Fig. 2a; Fig. S2B). In addition, 13 of the 422 genes were found to possess all four binding motifs (Table S1). Literature review for the correlations between any of these 13 genes and pathways related to tumorigenesis, KIT, or GISTs, we found NFKB, a NFKBIB-regulated protein, has been reported to mediate KIT expression in leukemia cells [14]. Therefore, we focused our studies on NFKBIB.

To validate the findings from our ChIP-seq analysis, ChIP assays were performed, and the data showed that KIT was bound to the *NFKBIB* promoter in all three GIST cell lines, and KIT binding to the *NFKBIB* promoter was reduced in *KIT*-silenced GIST48 and GIST430 cells (Fig. 2b**)**. Moreover, IM inhibited KIT binding to the *NFKBIB* promoter in GIST-T1 cells (Fig. S3). When KIT was silenced, both the protein and transcript levels of NFKBIB were reduced (Fig. 2c, d). Taken together, these data demonstrated that KIT bound to the *NFKBIB* promoter region and upregulated NFKBIB expression in GIST cells.

### Downregulation of NFKBIB leads to RELA activation, KIT reduction, and relative cell viability inhibition

**Fig. 3:**
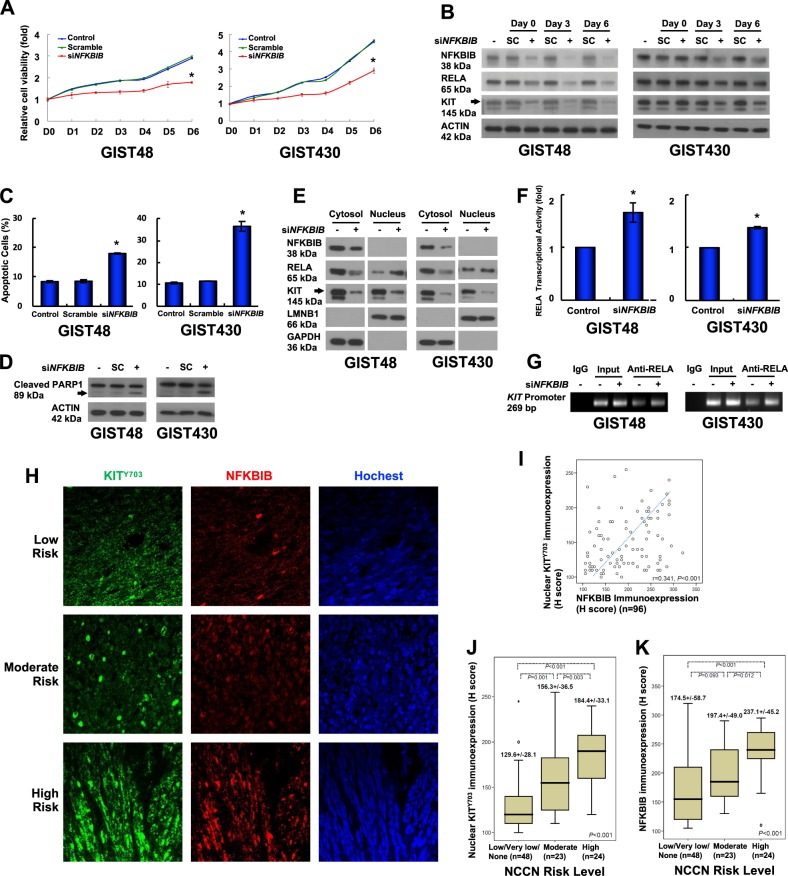
Role of KIT-regulated NFKBIB in GIST cell function. **a**, **b** GIST48 and GIST430 cells were transfected with 150 nM siRNA targeting *NFKBIB* or a scrambled control (SC) for 18 h. **a** The transfected cells were suspended, and equal numbers of cells were seeded into 24-well plates. After 6 h, the cells were attached and examined as the day 0 (D0) control, and the other cells were examined at the indicated times using a relative cell viability assay. **b** The cells were lysed and analyzed by immunoblotting. Actin served as an internal control. **c**–**g** Cells were transfected with siRNA targeting *NFKBIB* or a scrambled control for 72 h. The cells were analyzed by Annexin V staining (**c**) or immunoblotting against PARP1 (**d**). **e** The transfected cells were separated into cytoplasmic and nuclear fractions and analyzed by immunoblotting. LMNB1 and GAPDH were used as nuclear and cytoplasmic markers, respectively. **f** Nuclear proteins were used to analyze the RELA transcriptional activity. **g** Chromatin from the transfected cells was cross-linked, sheared, immunoprecipitated using an anti-RELA antibody, and amplified by PCR. Chromatin that was sheared but not immunoprecipitated was used as an input control. All experiments were repeated at least three times. **h** Representative GIST tissue samples were analyzed by immunostaining against p-KIT (KIT^Y703^; green) and NFKBIB (red) and visualized by confocal microscopy. Using photomicrographs, GISTs were classified as low-risk, moderate-risk, or high-risk according to NCCN consensus criteria based on increasing mitoses. **i** The scatter plot showed the correlations between 96 GIST tissues with various risk levels and the H-scores of the immunoexpression levels of nuclear KIT^Y703^ and NFKBIB. The box plot showed the associations between the nuclear KIT^Y703^ (**j**) and NFKBIB (**k**) immunoexpression levels and the H-scores for tumor grade based on the NCCN risk level according to the Kruskal–Wallis test. The middle line demonstrated the median, the box illustrated the interquartile range, and the whiskers indicated the extreme data points >1.5x the interquartile range from the box. *p < 0.05

NFKBIB, similar to NFKB inhibitor alpha (NFKBIA), is a cytoplasmic protein that binds to NFKB to inhibit its activity. However, NFKBIA and NFKBIB are responsible for the transient and persistent phases of NFKB activation, respectively [15]. To investigate the biological role of NFKBIB in KIT-expressing GIST cells, NFKBIB was downregulated using siRNA. As a result, relative cell viability was inhibited compared with untreated and scrambled control cells (Fig. 3a). In addition, downregulation of NFKBIB was associated with a consistent reduction of the protein levels of KIT and RELA, a subunit of NFKB, on day 3 and day 6 after siRNA treatment in GIST48 and GIST430 cells (Fig. 3b). The reduction of RELA could be resulted from its activation, nuclear translocation, and degradation in the nucleus, as previously reported [16]. Furthermore, NFKBIB downregulation induced apoptosis and increased the percentage of Annexin V-positive cells and PARP1 cleavage (Fig. 3c, d). These data showed that NFKBIB downregulation led to KIT reduction and apoptosis in GIST cells.

In response to NFKB stimulation, NFKBIA and NFKBIB are phosphorylated by IKK and then ubiquitinated and degraded, leading to the activation and nuclear translocation of NFKB, where it binds to its recognition sites in promoters and activates gene transcription [17, 18]. In this study, when NFKBIB was downregulated, RELA nuclear translocation and its transcriptional activity were enhanced (Fig. 3e, f). Consequently, KIT expression was reduced in both the cytoplasm and the nucleus (Fig. 3e). These findings indicated that RELA reduction in the cytoplasm (Fig. 3b) was associated with an increased level in the nucleus, as previously reported [16]. A previous study demonstrated that RELA could form a complex with SP1 and bind to the promoter region of *KIT* in myeloid leukemia cells [14]. We used ChIP assays to show the binding of RELA to the *KIT* promoter region in both GIST cells, and the binding was enhanced by NFKBIB silencing as compared with the untreated control cells (Fig. 3g). Taken together, these findings indicated that NFKBIB was crucial for KIT expression and cell survival in GIST cells.

Clinically, deidentified, surgically resected GIST tissues from 96 patients were used to evaluate the correlation among National Comprehensive Cancer Network (NCCN) risk, the expression levels of NFKBIB and nuclear phospho-KIT in GIST cells by immunofluorescence staining. Representative staining of KIT^Y703^ in the nucleus and the NFKBIB expression in GISTs of various NCCN risk was shown in Fig. 3h. By H-score estimation, NFKBIB expression was significantly correlated with that of nuclear KIT^Y703^ (Fig. 3i). Furthermore, the expression levels of either nuclear KIT^Y703^ or NFKBIB was significantly higher in high-risk GISTs as compared with those in moderate- and low-/very low-risk GISTs (Fig. 3j, k). These results suggested that the expression levels of NFKBIB and nuclear phospho-KIT were highly associated with tumorigenesis in GISTs.

### RELA overexpression leads to self-activation and reductions in KIT and relative cell viability of GIST cells

**Fig. 4:**
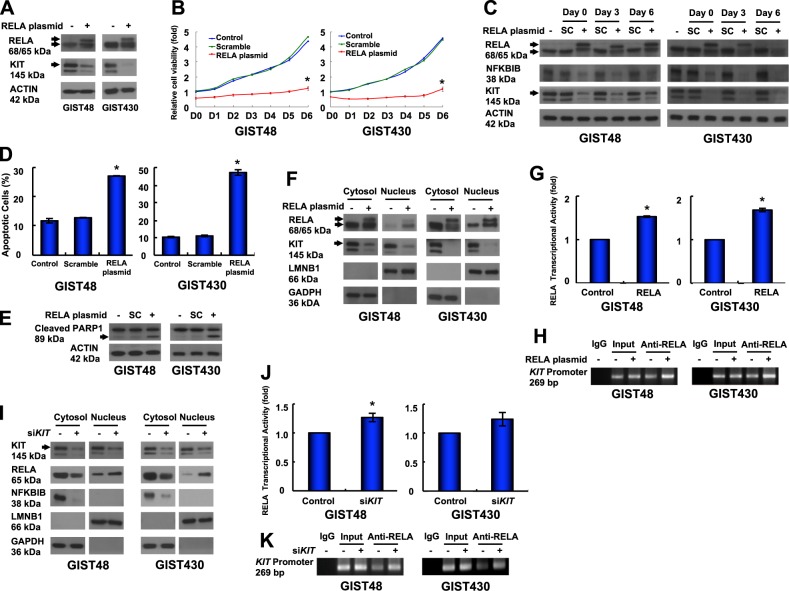
Roles of RELA in KIT expression and GIST cell function. **a** Cells were transfected with the *RELA*/pcDNA3.1 plasmid, lysed, and analyzed by immunoblotting against RELA and KIT. **b**–**h** The cells were transfected with *RELA*/pcDNA3.1 or a scrambled control (SC) for 18 h. The transfected cells were resuspended, and equal numbers of cells were seeded into 24-well plates. **b** After 6 h, the cells were attached and examined as the day 0 (D0) control, and the other cells were examined at the indicated times using a relative cell viability assay. **c** The cells were lysed and analyzed by immunoblotting. The transfected cells were analyzed by Annexin V staining (**d**) or immunoblotting against PARP1 (**e**). **f** The transfected cells were separated into cytoplasmic and nuclear protein fractions and analyzed by immunoblotting. **g** The nuclear protein fraction was analyzed to determine RELA transcriptional activity. **h** Chromatin obtained from the transfected cells was cross-linked, sheared, immunoprecipitated using an anti-RELA antibody, and amplified by PCR. Chromatin that was sheared but not immunoprecipitated was used as an input control. **i**–k Cells were transfected with siRNA targeting *KIT* or a scrambled control for 72 h. **i** The cells were separated into cytoplasmic and nuclear protein fractions and analyzed by immunoblotting. **j** The nuclear protein fraction was analyzed for RELA transcriptional activity. **k** Chromatin from the transfected cells was cross-linked, sheared, immunoprecipitated using an anti-RELA antibody, and amplified by PCR. Chromatin that was sheared but not immunoprecipitated was used as an input control. All experiments were repeated at least three times. The data are expressed as the means ± SD of two or more independent experiments. ACTIN (**a**, **c**, **e**) served as an internal control. LMNB1 and GAPDH (**f** and **i**) were used as nuclear and cytoplasmic markers, respectively. *p < 0.05

In this study, we found that KIT was reduced in *NFKBIB*-silenced, RELA-activated GIST cells. Thus, we further investigated the role of RELA in KIT expression and cell growth in GIST cells. The data showed that *RELA*/pcDNA3.1 plasmid-transfected cells expressed RELA in two forms (68 and 65 kDa) that led to a reduction in KIT expression (Fig. 4a). The sequence of the *RELA*/pcDNA3.1 plasmid was confirmed, and the data were validated using another antibody from a different clone (SC-8008, Santa Cruz; Fig. S4A). Moreover, KIT mRNA was reduced in RELA-overexpressing GIST cells (Fig. S4B). Relative cell viability was also inhibited in RELA-overexpressing GIST cells compared with untreated and scrambled controls (Fig. 4b). When RELA was overexpressed, the protein levels of KIT and NFKBIB were reduced on day 0, day 3, and day 6 (Fig. 4c). Furthermore, the percentage of Annexin V-positive cells and PARP1 cleavage were increased (Fig. 4d, e), indicating that RELA overexpression induced KIT reduction and apoptosis in GIST cells. In addition, the nuclear translocation and transcriptional activity of RELA were enhanced (Fig. 4f, g). Consequently, RELA binding to the *KIT* promoter was increased (Fig. 4h). These data demonstrated that RELA overexpression led to enhanced RELA activation and reduced KIT expression through transcriptional regulation. These results were consistent with the findings shown in Fig. 3. Furthermore, to further confirm the KIT-NFKBIB-RELA loop, KIT was downregulated by siRNA, which led to a reduction in NFKBIB expression in the cytoplasm and enhanced RELA nuclear translocation in GIST cells (Fig. 4i). In addition, RELA transcriptional activity and its binding to the *KIT* promoter were enhanced (Fig. 4j, k). Taken together, the above data confirmed the existence of a KIT-NFKBIB-RELA autoregulatory loop through which nuclear KIT upregulated NFKBIB expression to inhibit RELA activation and to enhance KIT expression in GIST cells.

### Valproic acid has antitumor activity via induction of RELA nuclear translocation, which leads to KIT downregulation

**Fig. 5:**
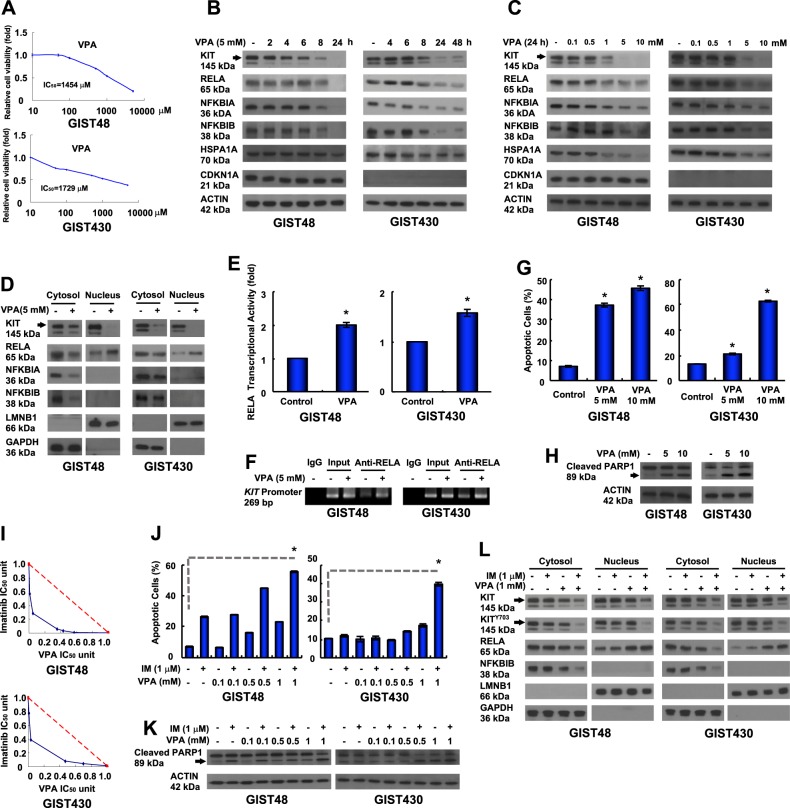
Antitumor activity of VPA was mediated by KIT downregulation, and relative cell viability was inhibited by RELA activation. **a** GIST48 and GIST430 cells were incubated with VPA at the indicated doses, and the IC_50_ was determined using a relative cell viability assay. **b**–**d** The cells were treated with 5 mM VPA for the indicated times (**b**), or treated with the indicated dose of VPA for 24 h (**c**). The total cell lysates or fractionated proteins (**d**) were extracted from the cells and analyzed by immunoblotting. **e**, **f** The cells were incubated with 5 mM VPA for 24 h. **e** The nuclear protein fraction was analyzed for RELA transcriptional activity. **f** Chromatin obtained from VPA-treated cells was cross-linked, sheared, immunoprecipitated using an anti-RELA antibody, and amplified by PCR. Chromatin that was sheared but not immunoprecipitated was used as an input control. **g**, **h** The cells were incubated with VPA and then analyzed by Annexin V staining (**g**) or immunoblotting against PARP1 (**h**). **i** Relative cell viability was determined using a methylene blue dye assay as described in the Supplementary Experimental Procedures. The interaction between VPA and IM at the IC_50_ value was analyzed using the isobologram method. **j**, **k** Cells were treated with various concentrations of VPA with or without IM and then analyzed by Annexin V staining (**j**) or immunoblotting against PARP1 (**k**). **l** Cells were treated with 1 mM VPA with or without 1 µM IM for 24 h. The fractionated proteins were extracted from the cells and analyzed by immunoblotting. The data were expressed as the means ± SD of three or more independent experiments. ACTIN (**b**, **c**, **h**, **k**) served as an internal control. LMNB1 and GAPDH (**d** and **l**) were used as nuclear and cytoplasmic markers, respectively. *p < 0.05

Based on our findings, NFKBIB inhibition and RELA activation are potential, alternative therapeutic strategies for IM-resistant, KIT-expressing GISTs. VPA is a clinically available drug for the treatment of epilepsy and bipolar disorder. VPA has been shown to block voltage-dependent sodium channels, to act as a histone deacetylase (HDAC) inhibitor, and to inhibit NFKB activation, which may affect the immune response [19]. Therefore, we examined the inhibitory effect of VPA on NFKB activity in IM-resistant GIST cells and a xenograft animal model. The data showed that VPA could inhibit the relative viability of GIST cells in vitro (Fig. 5a). VPA reduced the protein levels of NFKBIA, NFKBIB, and RELA in dose- and time-dependent manners in GIST48 and GIST430 cells (Fig. 5b, c). RELA expression was downregulated, and KIT expression was reduced in GIST48 and GIST430 cells treated with 5 mM VPA for 8 h and 24 h. Because VPA has been shown to inhibit HDAC activity in previous studies, we also measured HSPA1A and CDKN1A protein levels as indicators of HDAC inhibition. The data showed that neither HSPA1A nor CDKN1A was increased after treatment with 5 mM VPA for 24 h and 48 h, indicating that HDAC may not inhibit by VPA under these conditions in GIST cells. To further validate the inhibitory effects of VPA on protein kinases, additional protein kinase profiling assays were performed, and the data showed that the activity of 358 protein kinases remained at over 80% after VPA treatment (Table S2). Taken together, these results indicated that the VPA-induced downregulation of KIT was due to NFKB activation in IM-resistant GIST cells.

VPA at 5 mM reduced cytoplasmic NFKBIB, which enhanced RELA nuclear translocation and downregulated KIT in both the cytoplasm and the nucleus (Fig. 5d). The transcriptional activity of RELA and its binding to the *KIT* promoter were enhanced after GIST cells were exposed to 5 mM VPA for 24 h (Fig. 5e, f). Moreover, KIT mRNA was reduced in VPA-treated GIST cells (Fig. S5). Furthermore, 5 mM and 10 mM VPA increased the percentage of Annexin V-positive cells and PARP1 cleavage in GIST cells (Fig. 5g, h). Conclusively, similar to the effects of NFKBIB silencing or RELA overexpression, treatment with VPA reduced NFKBIB expression, which led to enhanced RELA activation, and binding to the promoter region of *KIT*. Consequently, VPA reduced KIT expression and enhanced apoptosis in IM-resistant GIST cells.

Although VPA could activate RELA and downregulate KIT, the dose requirement of 5 mM VPA is higher than its steady-state serum concentration of 0.86 mM. Thus, to reduce the dose requirement of VPA for future clinical approaches, the combined inhibitory effects of IM and VPA on the phospho-KIT expression level and GIST cell growth were examined. IM and VPA co-treatment had a better inhibitory effect on the viability of IM-resistant GIST48 and GIST 430 cells than each treatment alone (Fig. 5i). Furthermore, 1 μM IM combined with 1 mM VPA increased the percentage of Annexin V-positive cells and PARP cleavage of GIST cells over that observed in the control or cells treated with individual agent alone (Fig. 5j, k). In addition, combining 1 μM IM with 1 mM VPA reduced cytoplasmic NFKBIB and enhanced RELA nuclear translocation that was accompanied with decreased expression levels of the total KIT and phospho-KIT in both the cytoplasm and the nucleus (Fig. 5l). Taken together, these data indicated that the combination of IM and VPA showed better inhibitory effects on phospho-KIT levels and enhanced apoptosis in IM-resistant GIST cells.

### A combination of IM and low-dose VPA achieves tumor growth inhibition comparable with that of high-dose VPA

**Fig. 6:**
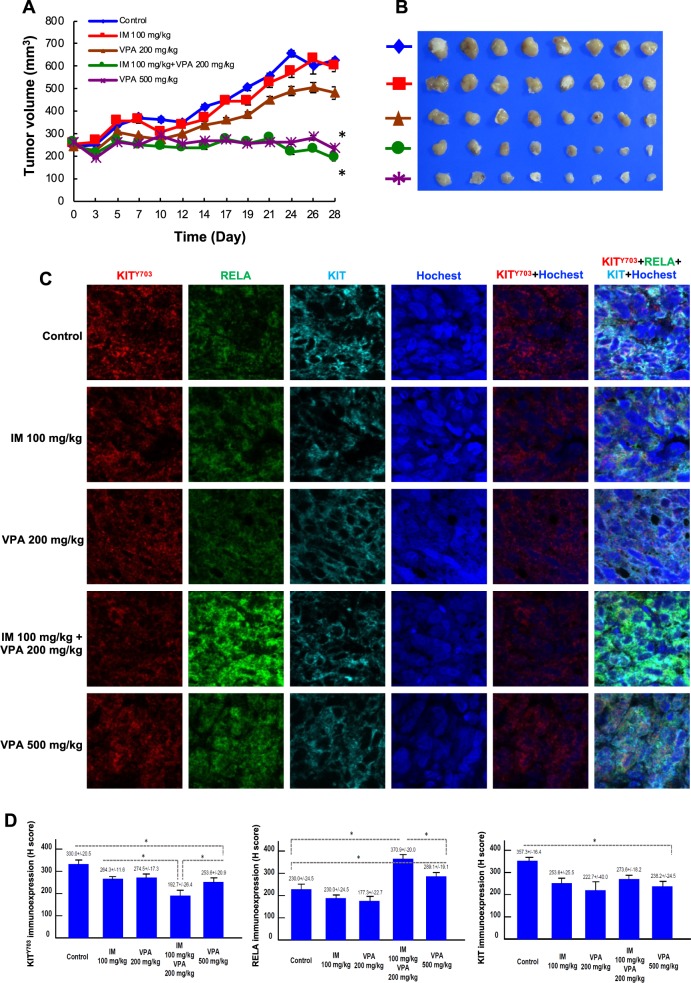
Effect of VPA either alone or combined with IM in a GIST430 xenograft animal model. **a**–**d** GIST430 xenografts were established as described in the Materials and Methods section. Mice received an intraperitoneal injection of control (DMSO), 100 mg/kg IM, 200 mg/kg VPA, 100 mg/kg IM with 200 mg/kg VPA, and 500 mg/kg VPA, were administered i.p. twice per week for 4 weeks (*n* = 8/group). **a** Tumor volume was calculated as 1/2 x length x width^2^. **b** The tumors were collected at the end of drug administration and analyzed by immunostaining (**c**) against KIT^703^, RELA, or KIT. The immunoexpression levels were quantified (**d**). The data are expressed as the means ± SD of three or more independent experiments. *p < 0.001

The inhibitory effects of IM with low-dose VPA in inhibiting GIST cell growth in vitro was further evaluated in a GIST430 xenograft animal model in vivo. Based on previous studies of VPA in other xenograft models, the in vivo VPA concentrations of 200 mg/kg (low-dose) and 500 mg/kg (high-dose) were converted to 1 mM and 5 mM VPA, respectively, in vitro [20–22]. Therefore, five treatment groups, including control (DMSO), 100 mg/kg IM, 200 mg/kg VPA, 100 mg/kg IM with 200 mg/kg VPA, and 500 mg/kg VPA, were administered i.p. twice per week for 4 weeks. The mean tumor volumes in the corresponding groups of mice were 624.85, 597.38, 481.53, 189.91, and 234.91 mm^3^, respectively (Fig. 6a, b). High-dose VPA effectively inhibited tumor growth, similar to the effects of 5 mM VPA in GIST cells in vitro. Moreover, the tumor volumes at the end of treatment in the mice co-treated with IM and low-dose VPA were significantly smaller than those of mice treated with IM alone or low-dose VPA alone. Compared with the control group, mice administered high-dose VPA or IM combined with low-dose VPA had significantly smaller tumors on day 28 (*p* < 0.001 and *p* < 0.001, respectively). There was no significant difference in mean tumor volume between the mice treated with high-dose VPA and those treated with IM combined with low-dose VPA. The KIT^Y703^ immunoexpression levels in the GIST430 xenografts were significantly reduced in the high-dose VPA group and the IM combined with low-dose VPA group compared with the control group (Fig. 6c, d). Moreover, the immunoexpression levels of the total KIT were lower in tumor tissues obtained from the groups treated with high-dose VPA or IM combined with low-dose VPA than in the control group. Taken together, these data showed that IM combined with low-dose VPA reduced phospho-KIT and total KIT expression levels and had inhibitory effects on tumor growth in a GIST430 animal xenograft. These effects were comparable with those of high-dose VPA. These findings indicated that targeting NFKB with VPA represents a potential therapeutic strategy for IM-resistant, KIT-expressing GISTs.

## Discussion

**Fig. 7:**
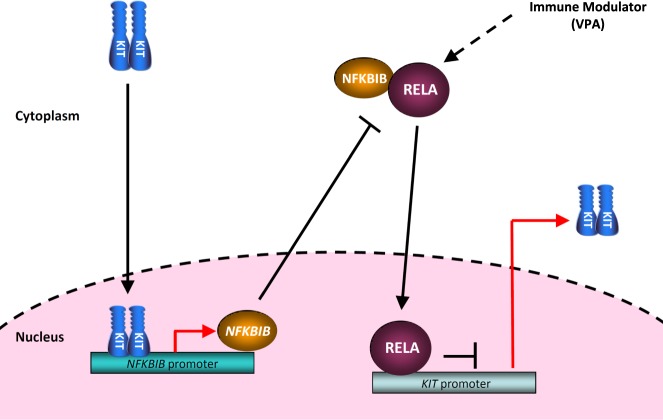
Schematic of the KIT-NFKBIB-RELA autoactivation loop in mutant KIT-expressing GIST cells

This is the first study to demonstrate that KIT can be in the nucleus and regulate gene expression in KIT-expressing GIST cells. A schematic summary of our findings is shown in Fig. 7. In GIST cells, mutated and phosphorylated KIT in the nucleus bound to the *NFKBIB* promoter, and drove NFKBIB expression, leading to the inactivation of NFKB, which blocked the RELA-mediated inhibition of *KIT* transcription. Moreover, treatment with VPA enhanced the nuclear translocation and binding of RELA to the *KIT* promoter, which led to KIT downregulation and inhibited relative cell viability. A serum-achievable dose of VPA with IM had significant inhibitory effects in IM-resistant GIST cells and a GIST430 xenograft animal model. The mechanisms underlying nuclear KIT and the NFKBIB-RELA-KIT pathway involved an autoregulatory loop mediating KIT expression and supported the notion that NFKBIB and NFKB are therapeutic targets for KIT-expressing GISTs. In addition, these results suggested that targeting NFKB with VPA represents a potential therapeutic strategy for reducing KIT phosphorylation and expression in IM-resistant GIST patients.

The mechanism underlying IM resistance involves a gain-of-function secondary mutation in exons 13, 14, or 17 of KIT. Secondary mutations in KIT in exons 13/14 and 17 lead to conformational changes into the intermediate and fully active forms, respectively [23–25]. These conformational changes produce steric hindrance, which reduces the binding of IM to the ATP-binding pocket. Therefore, secondary mutations of KIT in exon 13/14 or 17 are responsible for different kinase conformations that may be compatible with drugs with different structures. Downregulating KIT with a siRNA or HSP90AA1 inhibitors effectively reduced phosphorylated KIT and downstream signaling, which induced cell death and tumor shrinkage in IM-resistant GIST48 and GIST430 cells [12, 13, 26]. A MTOR inhibitor and autophagy inducer, such as rapamycin, could enhance autophagy, leading to reductions in total and phospho-KIT expression levels in IM-resistant GIST cells [12]. Furthermore, bortezomiband-specific antibodies (2D1 and 3G1-Fc) against KIT also induced KIT internalization and degradation [27, 28]. Therefore, the development of a potent drug to downregulate KIT expression would represent an alternative therapy for TKI-resistant GIST regardless of the KIT mutation sites. In this study, we identified a NFKBIB-RELA-KIT autoregulatory loop that mediates KIT expression and potential therapeutic targets that downregulate KIT expression levels and may be useful for the treatment of IM-resistant GISTs.

Although previous evidence indicated that aberrant inflammation, partially through NFKB activation, was responsible for the initiation of tumorigenesis, recent findings have demonstrated that NFKB activation inhibits carcinogenesis and tumorigenesis [29, 30]. The first evidence directly linking NFKB to tumor suppression was found in the epidermis overexpressing the NFKB inhibitory protein NFKBIA [29]. Functional blockade of NFKB in epidermal cells resulted in severe hyperplasia of the skin in the transgenic mice, and effect that could be reversed by the overexpression of active RELA and the NFKB1 subunit of NFKB, suggesting that NFKB has a tumor suppressive effect [30]. Deficiency in the *IKBKB* gene, an activator of NFKB, promoted the migration and proliferation of mouse embryo fibroblasts [31]. Mice lacking IKBKB in hepatocytes exhibited a marked increase in diethylnitrosamine-induced hepatocarcinogenesis [32]. The deletion of NEMO/IKBKG, an activation complex for NFKB, in liver parenchymal cells led to steatohepatitis and hepatocellular carcinoma [33]. These studies indicate that NFKB activation provides inhibitory signals for tumor growth and tumorigenesis, similar to our findings in GIST cells. In summary, the functional outcome of NFKB (tumor suppression or tumor promotion) may be determined by the cell type, the stimulator, the duration of activation, and other accompanying signals.

In addition to NFKBIB, 12 other genes harbor four potential KIT-binding motifs (Table S1). A review of the literature revealed that aberrant expression of CKMT1A, GCNT1, NCALD, NFKBIB, NOMO3/Nodal, SLC16A5, or TRPV2 was correlated with cancer [34–41]. Although few reports have investigated the roles of these genes in cancer development and metastasis, none of the 12 genes containing KIT-binding motifs are directly correlated with KIT or GISTs. Therefore, further research on the KIT-binding promoters of these 12 genes containing all four binding motifs may help clarify nuclear KIT-related tumorigenesis in GISTs. Furthermore, nuclear EGFR and IGF1R have both been shown to interact with conventional transcription factors to regulate gene expression. Therefore, we suggest that KIT binding to the *NFKBIB* promoter may also involve a binding partner. Additional studies will be needed to identify the binding partner of nuclear KIT.

There are some limitations to this study. First, our data only explain the mechanisms of KIT-related tumorigenesis in GISTs. Worldwide, there are only five patient-derived GIST cell lines, and only GIST48 and GIST430 parental cell lines are resistant to IM. Moreover, GIST48 and GIST430 cells harbor KIT mutations in exon 11/17 and exon 11/13, respectively, and are responsible for over 50% of IM-resistant cases of GIST. Therefore, our in vitro findings based on GIST48 and GIST430 cells are representative phenomena observed in IM-resistant, KIT-expressing GISTs. Second, VPA is a multifunctional drug that may play an alternative role to cause KIT downregulation and GIST cells death. Third, only one GIST xenograft model (GIST430) was used in our study, although we also tried to establish xenograft models of GIST48, and GIST PDX. In a previous study, we demonstrated the antitumor activity of NVP-AUY922 using a GIST430 xenograft model, and a further phase I/II clinical trial has confirmed our findings [12], indicating that the GIST430 xenograft model is a suitable preclinical model for further preparing for a clinical trial [42].

In summary, this is the first report to demonstrate that nuclear KIT could regulate the expression of genes such as NFKBIB and form a positive KIT-NFKBIB-RELA autoregulatory loop that mediates KIT expression, which has been correlated with KIT-related tumorigenesis in GISTs. These findings not only clarify the mechanisms underlying KIT-related tumorigenesis in GISTs but also suggest that NFKBIB and RELA are therapeutic targets for IM-resistant, KIT-expressing GISTs.

## Materials and methods

Additional details of reagents, ChIP assay, ChIP-seq and binding motif analysis, transient transfection, RNA interference, protein fractionation, relative cell viability assay, RNA extraction and quantitative analysis of mRNA, RELA transcriptional activity assay, and protein kinase profiling were described in the Supplementary Experimental Procedures.

### Cell lines and plasmids

GIST430 and GIST48 cells encoding exon 11^V560_L576del^/13^V654A^ and exon 11^V560D^/17^D820A^ mutant KIT oncoproteins, respectively, were maintained as previous studies [12, 13]. Cells were authenticated by *KIT* sequencing and KIT TKI sensitivity experiments. Cells were tested for mycoplasma contamination by the PCR mycoplasma detection kit (Applied Biological Materials, Canada) and were found to be negative. GIST-T1 cells with exon 11^V560-Y578del^ mutant KIT [43] and COS-1 cells were obtained from COSMO BIO (PMC-GIST01-COS, Japan) and BCRC (#60002, Taiwan), respectively. The *RELA*/pcDNA3.1 plasmid obtained from Dr. Hung (Wen-Chun, National Health Research Institutes [NHRI], Taiwan) was sequenced.

### Immunofluorescence staining of GIST cells

Detailed protocol was described previously [12], and the antibodies used are listed in Table 1.

**Table 1:** Antibodies Used in Human and Mouse Immunostaining studies

Target	Supplier	Host	Clone/ID	Isotype	Label	Final concentration
KIT	Dako	Rabbit pAb	A4502			1:500
LMNB1	SCBT	Goat pAb	SC-6216			1:100
p-KIT (clinical sample and mouse)	CST	Rabbit pAb	3073 S			1:1000
NFKBIB (clinical sample and mouse)	Abcam	Rabbit pAb	ab109509			1:1000
RELA (clinical sample and mouse)	Abcam	Rabbit pAb	ab76311			1:1000
Secondary Ab: anti-rabbit IgG (H + L)	JIR	Goat pAb	111–225–144		Cy^Tm^2	1:100
Secondary Ab: anti-goat IgG (H + L)	JIR	Donkey pAb	705–025–147		Rhodamine	1:100
Secondary Ab: (for clinical sample and mouse): anti-mouse	Dako	Rabbit–mouse	k5007		Envision-HRP	1:1

*H* *+* *L* highly cross-adsorbed, *mAb* monoclonal antibody, *pAb* polyclonal antibody

Abcam, Inc, Cambridge, UK; CST (Cell Signaling Technology, Inc.), Beverly, MA; Dako, Inc., Carpinteria, CA; JIR (Jackson ImmunoResearch, Inc.) West Grove, PA; SCBT (Santa Cruz Biotechology, Inc.) Dallas, TX

### Chromatin immunoprecipitation (ChIP) assay

ChIP analysis was performed using an EZ-Magna ChIP G kit (Merck KGaA, Darmstadt, Germany) according to the manufacturer’s protocol. Detailed protocol was described in the Supplementary Experimental Procedures, and the primers used were listed in Table S3.

### RNA extraction and quantitative analysis of mRNA

Detailed protocol was described in the Supplementary Experimental Procedures, and the primers used are listed in Table S4.

### Immunoblotting

Detailed protocol was described previously [12], and the antibodies used were listed in Table 2.

**Table 2:** Antibodies Used in Immunoblotting studies

Target	Supplier	Host	Clone/ID	Isotype	Label	Final concentration
KIT	Dako	Rabbit pAb	A4502			1:2500
KIT^Y703^	CST	Rabbit pAb	#3073			1:1000
NFKBIA	CST	Rabbit pAb	#9242			1:1000
NFKBIB	GeneTex	Mouse mAb	GTX82797	IgG1		1:1000
RELA	CST	Mouse mAb	#6956	IgG2b		1:1000
PARP1	CST	Rabbit pAb	#9542			1:1000
HSPA1A	Enzo	Mouse Ab	ADI-SPA-810-F	IgG1		1:1000
CDKN1A	CST	Rabbit pAb	#2947			1:1000
LMNB1	SCBT	Goat pAb	SC-6216			1:1000
GAPDH	SCBT	Rabbit pAb	SC-25778			1:1000
ACTIN	Merck KGaA	Mouse mAb	MAB1501	IgG1k		1:10,000
Secondary Ab: anti-rabbit IgG (H + L)	JIR	Donkey pAb	711–035–152		HRP	1:20000
Secondary Ab: anti-mouse IgG (H + L)	JIR	Donkey pAb	715–035–150		HRP	1:20,000
Secondary Ab: anti-goat IgG (H + L)	JIR	Donkey pAb	705–035–147		HRP	1:20,000

*H* *+* *L* highly cross-adsorbed, *mAb* monoclonal antibody, *pAb* polyclonal antibody, *HRP* horseradish peroxidase

CST (Cell Signaling Technology, Inc.), Beverly, MA; Dako, Inc., Carpinteria, CA; Enzo Life Sciences, Inc, Farmingdale, NY; GeneTex Inc., Hsinchu, Taiwan; JIR (Jackson ImmunoResearch, Inc.) West Grove, PA; SCBT (Santa Cruz Biotechology, Inc.) Dallas, TX; Merck KGaA, Darmstadt, Germany

### GIST430 xenograft animal model and drug treatment

All procedures using animals were approved by the Institutional Animal Care and Use Committee of the NHRI (NHRI-IACUC-104057-AP). Eight-week-old male NOD/SCID mice were purchased from LASCO (Taipei, Taiwan). A total of 2 × 10^7^ GIST430 cells were mixed with an equal volume of Matrigel (BD Biosciences), and each mouse was subcutaneously implanted with 0.1 ml of the cell-Matrigel mixture in one flank. The tumor volume was estimated using caliper measurements twice per week after implantation, and was calculated as 1/2 × length × width^2^ [44]. When the tumors had developed for ~3 weeks and tumor volume had reached 200–300 mm^3^, the mice were randomized to five groups and administrated an intraperitoneal injection of control (DMSO), 100 mg/kg IM, 200 mg/kg VPA, 100 mg/kg IM with 200 mg/kg VPA, and 500 mg/kg VPA, respectively, twice per week for 4 consecutive weeks (*n* = 8/group). Tumor volume was measured twice per week after drug treatment. At the end of the experiments, the animals were killed, and the tumors were visualized and then stored at −80 °C for further analyses.

### Immunofluorescence staining of tumor specimens

The protocol using human tissue blocks was approved by the Institutional Review Board of National Cheng Kung University Hospital (AER-104–074) and Chi-Mei Foundation Medical Center (1050101). Sections of tumor tissues obtained from GIST patients or the GIST430 xenograft animal model were cut at 3-μm thick, and placed on an adhesive-coated glass slide system (Instrumedics, Hackensack, NJ). To overcome to slow penetration of formalin which prohibits the preservation of phosphoproteins, selective tumors samples were cut into slices ~2-μm thick and fixed in formalin immediately after tumor removal. After a 24-h fixation, tissue was paraffin embedded and sliced. The slides were dewaxed in xylene and rehydrated through an alcohol gradient down to water. Then, the slides were pressure cooked in 10 mM citrate buffer at pH 6 for 7 min for antigen retrieval, and washed using TBS buffer with 0.1% Tween 80 for 5 min. The endogenous peroxidase activity was blocked by treatment with 3% H_2_O_2_. After they were washed, the slides were incubated with primary antibodies (see antibodies in Table 1). The slides were washed with PBS, incubated with a fluorescently labeled secondary antibody, washed with PBS, and examined under a confocal microscope. The slides were then counterstained with hematoxylin. Incubation without the primary antibody was performed for the negative control samples.

The slides were read visually, and the immunoexpression levels of nuclear phospho-KIT (KIT^Y703^) and NFKBIB were independently determined by one expert pathologist (CF Li) using the H-score method as previously described [45, 46] and defined by the equation Σ*Pi* (*i* + 1), where *i* is the intensity of the stained tumor cells (0–3^+^) and *Pi* is the percentage of stained tumor cells (ranging from 0 to 100%). The immunoexpression levels were dichotomized into low-/very low-risk, moderate-risk, and high-risk groups, and the cutoff was defined as the median value of individual averaged triplicate H-scores of 96 informative cases.

### Statistical analyses

We analyzed the data with the Statistical Package for the Social Sciences [Software version 16 for Windows (SPSS, Inc.)]. All data were expressed as the means ± SD. A comparison of the means among groups was performed using a one-way analysis of variance (ANOVA) followed by a Bonferroni post hoc test. The level of significance was set at *p* < 0.05.

## Supplementary information

Supplementary Experimental Procedures.

Supplementary TableS1.

Supplementary TableS2.

Supplementary TableS3.

Supplementary TableS4.

Supplementary FigureS1.

Supplementary FigureS2.

Supplementary FigureS3.

Supplementary FigureS4.

Supplementary FigureS5.
