# Supplementary material for: Nuclear KIT induces a NFKBIB-RELA-KIT autoregulatory loop in imatinib-resistant gastrointestinal stromal tumors
Source: Oncogene. 2019 Jul 30;38(38):6550–65. doi: 10.1038/s41388-019-0900-9 (PMC6756115; doi:10.1038/s41388-019-0900-9)
Supplement: Supplementary file 1 — Supplementary Experimental Procedures. [file 41388_2019_900_MOESM1_ESM.docx]

**Supplementary Experimental Procedures**

**Reagents**

IM was kindly supplied by Novartis. VPA was obtained from Cayman Chemical (Ann Arbor, MI). Sodium orthovanadate, sodium fluoride, β-glycerophosphate, PMSF, and CelLytic^TM^ cell lysis reagents were purchased from Sigma-Aldrich (St Louis, MO).

**Chromatin immunoprecipitation (ChIP) assay**

ChIP analysis was performed using an EZ-Magna ChIP G kit (Merck KGaA, Darmstadt, Germany) according to the manufacturer's protocol. Briefly, cells were treated with formaldehyde, and the cross-linked chromatin was digested enzymatically and sonicated to shear the cross-linked DNA for ChIP and ChIP-seq. The samples were precleared with magnetic Protein G beads and incubated with 5 μg of antibodies specific for KIT (Dako, Carpinteria, CA) and RELA (Cell Signaling Technology, Beverly, MA) or a normal IgG antibody (as a negative control) to assess nonspecific antibody binding. Immunocomplexes were precipitated using magnetic Protein G beads. After the samples were washed, eluted, and reverse cross-linked, the DNA fragments were isolated and purified with columns. For the ChIP assay, PCR was performed to amplify the promoter regions of *NFKBIB* and *KIT*, and the amplicons were analyzed by electrophoresis. The primers are listed in **Table S3**.

**ChIP-seq and binding motif analysis**

Purified ChIP DNA was used to prepare fragment libraries, which were subsequently analyzed by high-throughput sequencing using a SOLiD^TM^ 5500xl sequencer (Thermo Fisher Scientific, Inc., Waltham, MA). A total of 24–29 million raw reads were obtained for both normal rabbit IgG-precipitated and anti-KIT antibody-precipitated DNA. The raw reads were further analyzed using LifeScope^TM^ Genomic Analysis Software (version 2.5) and mapped to a human reference genome (hg19) in the UCSC database. To identify significant peaks, the mapped profiles were analyzed using the ChIP-seq tool in the CLC Genomics Workbench (version 4.9). The window size and false discovery rate were set at 200 bp and 5%, respectively. To determine high-confidence KIT binding loci, the ChIP region was identified by scanning the peaks for which read counts were significantly higher in the KIT-precipitated group than in the normal IgG-precipitated group. For the *de novo* motif discovery algorithm, the top 150 ranked KIT ChIP-seq peaks (±100 bp from the center of the ChIP-seq peaks) were analyzed using MEME software with the recommended default settings [1].

**Transient transfection**

All recombinant DNA studies were approved by the Institutional Biosafety Committee of the NHRI (IBC-104041). Transfection was performed using Lipofectamine 2000® according to the manufacturer’s protocol (Invitrogen, Carlsbad, CA). Briefly, cells at approximately 90% confluency were incubated with 2 μg of *RELA*/pcDNA3.1 plasmid and 2 μl of Lipofectamine 2000^®^ for 6 h. The transfected cells were used for further experiments after they were recovered via incubation in growth medium for 18 h.

**RNA interference**

All recombinant DNA studies were approved by the Institutional Biosafety Committee of the NHRI (IBC-104041). Small interfering RNAs (siRNAs) targeting human *NFKBIB* (HSS143106, HSS143106, and HSS181515) and *KIT* (HSS105820, HSS105821, and HSS105822) and the negative control siRNA were purchased from Invitrogen. Following the manufacturer’s protocol for Lipofectamine 2000®, cells at 90% confluency were transfected with 150 nM of annealed RNA duplexes mixed with 2 μl of Lipofectamine 2000^®^ for 6 h. After transfection, growth medium containing 20% FBS was added, and the cells were incubated for an additional 12 h or 66 h for each experiment.

**Protein fractionation**

Cells were grown to approximately 90% confluency. After incubation with drugs at the indicated doses for the indicated times, cytoplasmic proteins and nuclear proteins were extracted using buffer I (10 mM Tris-HCl pH 7.9, 10 mM KCl, 0.1 mM EDTA, 0.1 mM EGTA, and 1 mM DTT) and buffer II (20 mM Tris-HCl pH 7.9, 400 mM NaCl, 1 mM EDTA, 1 mM EGTA, and 1 mM DTT), respectively. Both buffers were supplemented with a protease inhibitor cocktail (Sigma-Aldrich), PMSF, and phosphatase inhibitors (sodium orthovanadate, sodium fluoride, and β-glycerophosphate). GAPDH and LMNB1 were used as cytoplasmic and nuclear markers, respectively.

**Relative cell viability assay**

For this assay, 4×10^4^ GIST48 or 5×10^4^ GIST430 cells that were left untreated or transfected were seeded in each well of a 24-well plate. The cells were incubated with or without drugs for the indicated number of days. A methylene blue dye assay was used to evaluate the effect of the drugs, protein overexpression, or gene downregulation on the relative number of viable cells. The data were obtained with a SpectraMax M5 microplate reader (Molecular Devices, Sunnyvale, CA) at 595 nm and normalized using the DMSO-only control group. The IC_50_ values were determined by plotting the growth relative to that of the untreated controls. All experimental points were measured in duplicate wells for each plate and replicated in at least three plates.

For analysis of the interaction between VPA and IM, the combination index (CI) was analyzed following previous study [2]. Briefly, the constant ratio combination design was chosen to assess the combined effect of both the drugs. Dose-response curves were determined with both drugs in combination at a fixed ratio that was equivalent to the ratio of their IC_50_ values. The CI value indicates if the effects of the two drugs were additive (CI≈1), synergistic (CI<1), or antagonistic (CI>1). All experimental points were measured in duplicate wells for each plate and were replicated in at least three plates.

**RNA extraction and quantitative analysis of mRNA**

RNA was isolated from cells using the RNeasy Mini Kit (QIAGEN, Hilden, Germany) according to the manufacturer’s instructions. Reverse transcription PCR was performed using ReverTra Ace (TOYOBO, Osaka, Japan). The cDNA expression of *KIT* and *NFKBIB* was quantified with the LightCycler TaqMan Master Kit (Roche Diagnostics) using a LightCycler Instrument (Roche Diagnostics, LightCycler System, Basel, Switzerland). The primers were designed using the Roche Universal ProbeLibrary and are listed in **Table S4**. The cycling protocol consisted of 95°C for 10 min followed by 45 cycles of 95°C for 10 seconds, 60°C for 30 seconds, and 72°C for 1 second. The amplification signals were detected in real time. To normalize the differences in the amount of total RNA added to each reaction, *ACTIN* was simultaneously processed in the same samples as an internal control. The mRNA levels of *KIT* and *NFKBIB* were determined as relative ratios, which were calculated by dividing the level of *KIT* or *NFKBIB* mRNA by the level of the *ACTIN* housekeeping gene from the same sample. The relative ratios of untreated cells were assumed to be 1, and the other samples were compared to the untreated controls. Each experiment was performed at least twice, and the data are expressed as the means ± S.D.

**RELA transcriptional activity assay**

The RELA transcriptional activity assay was performed according to the manufacturer's protocol (Cayman, Ann Arbor, MI). Briefly, equal amounts of nuclear proteins extracted from cells were added to each well of a 96-well plate, and 10 μl of positive protein with or without 10 μl of competitor dsDNA was included as a competitor control and a positive control, respectively. After incubation at 4°C overnight, each sample was washed and incubated with the anti-RELA primary antibody for 1 h at room temperature and then incubated with the secondary antibody. After the samples were incubated with the developing solution for 1 h, the reaction was inhibited with stop solution; then, the OD of the RELA-DNA complexes was detected at 450 nm. The relative ratios of untreated cells were assumed to be 1, and the other samples were compared to the untreated controls. All experimental points were measured in duplicate wells for each plate and replicated in at least three plates.

**Protein kinase profiling**

Protein kinase assays were conducted using the KinaseProfiler^TM^ service of Eurofins Pharma Discovery Services UK Limited (Dundee, United Kingdom). The kinase of interest was incubated with the test compound in assay buffer containing substrate, 10 mM magnesium acetate and [γ-^33^P]-ATP. The reaction was initiated by the addition of the Mg/ATP mix. After incubation at room temperature, the reaction was stopped by the addition of a 3% phosphoric acid solution. An aliquot of the reaction was then spotted onto a filter mat, which was washed in phosphoric acid and rinsed in methanol prior to drying and scintillation counting. The results were expressed in relation to controls containing DMSO only in place of the test compound. The ATP concentration in each assay was within 15 µM of the determined apparent *K_m_* for ATP.

**Supplementary References**

1. Bailey, T.L. and C. Elkan, *Fitting a mixture model by expectation maximization to discover motifs in biopolymers.* Proc Int Conf Intell Syst Mol Biol, 1994. 2: p. 28-36.
2. Hsueh, Y.S., et al., *Autophagy is involved in endogenous and NVP-AUY922-induced KIT degradation in gastrointestinal stromal tumors.* Autophagy, 2013. 9(2): p. 220-33.

**Supplementary Figure Legends**

**Figure S1. The z stack for images of GIST48 and GIST430 cells.**

Cells were stained using KIT and LMNB1 antibodies. After the cells were immunostained, they were visualized by confocal microscopy, and the images were acquired through the Cy2, Rhodamine, and DAPI channels (1000x). The Z stack for images of cells were dissected every 1 μm. The data were derived from representative images of 5 fields/picture for each sample.

**Figure S2. The binding motifs of shuffled background correlated to KIT-bound DNA.**

Chromatin from GIST48 cells was cross-linked, sheared, immunoprecipitated using a KIT antibody, and analyzed using next-generation sequencing (NGS). (**A**) Short reads obtained from NGS were mapped to the reference genome. Enriched reads (compared to a normal IgG-immunoprecipitated control) were adjusted using filters, and their distribution in the genome was determined. (**B**) The enriched reads from KIT-bound specific ChIP-seq peaks were randomized. Logos were obtained by running MEME-ChIP with 300-bp summits. The numbers next to the logos indicate the occurrence of the motifs and the statistical significance (E-value).

**Figure S3. The binding of KIT to *NFKBIB* promoter in GIST-T1 cells.**

Cells were treated with 1 μM IM for 8h. The chromatin was cross-linked, sheared, immunoprecipitated using a KIT antibody, and amplified by PCR. Chromatin that was sheared but not immunoprecipitated was used as an input control. All experiments were repeated at least three times.

**Figure S4. The role of RELA in KIT expression of GISTcells.**

(**A**) The cells were transfected with the RELA/pcDNA3.1 plasmid, lysed, and analyzed by immunoblotting against RELA using another antibody with different clone (SC-8008, Santa Cruz). (**B**) The RNA extracted from *RELA*-overexpressing cells were analyzed by real-time PCR. Actin served as an internal control for both protein and RNA loading. All experiments were repeated at least three times.

**Figure S5. The RNA level of KIT after VPA treatment in GIST cells.**

GIST48 and GIST430 cells were incubated with 5 mM VPA for 48 h. The RNA was extracted and analyzed by real-time PCR. Actin served as an internal control for RNA loading. All experiments were repeated at least three times.
