# Supplementary material for: Nuclear KIT induces a NFKBIB-RELA-KIT autoregulatory loop in imatinib-resistant gastrointestinal stromal tumors
Source: Oncogene. 2019 Jul 30;38(38):6550–65. doi: 10.1038/s41388-019-0900-9 (PMC6756115; doi:10.1038/s41388-019-0900-9)
Supplement: Supplementary file 2 — Supplementary TableS1. [file 41388_2019_900_MOESM2_ESM.pdf]

Table S1. Genes involved in the subsets of 4 binding motifs

Genes involved in 1 of 4 binding motifs

AK4	ANLH	ARH6EF15	BCRP2	CCDC108
DNMT1	PCDH6B6	PPP1R42	PRR52	TGEF1
TGIF1	TSPAN10	ZNF498		

Genes involved in 2 of 4 binding motifs

BBS2	JAK3	REREP3	FOXRED2	

Genes involved in 3 of 4 binding motifs

C9orf24	DDX56	FABP6	LCP2	LRCH3
MME	RNF10	ZNF14		

Genes involved in 4 of 4 binding motifs

CKMT1A	FA2H	GARS	GCNT1	NCALD
NFKBIB	NOMO3	OR7G2	SLC16A5	SPDYE2L
TMOD3	TRPV2	VPS37D		
