# Supplementary material for: Nuclear KIT induces a NFKBIB-RELA-KIT autoregulatory loop in imatinib-resistant gastrointestinal stromal tumors
Source: Oncogene. 2019 Jul 30;38(38):6550–65. doi: 10.1038/s41388-019-0900-9 (PMC6756115; doi:10.1038/s41388-019-0900-9)
Supplement: Supplementary file 3 — Supplementary TableS2. [file 41388_2019_900_MOESM3_ESM.pdf]

Table S2. Protein kinase activities after VPA treatment

Kinase	Activity (% Control)	Kinase	Activity (% Control)
Abl(h)	101	CaMKK1(h)	92
Abl (H396P) (h)	102	CaMKK2(h)	98
Abl (M351T)(h)	90	CDK1/cyclinB(h)	105
Abl (Q252H) (h)	90	CDK2/cyclinA(h)	104
Abl(T315I)(h)	107	CDK2/cyclinE(h)	109
Abl(Y253F)(h)	106	CDK3/cyclinE(h)	109
ACK1(h)	97	CDK4/cyclinD3(h)	107
ALK(h)	94	CDK5/p25(h)	96
ALK1(h)	110	CDK5/p35(h)	111
ALK2(h)	103	CDK6/cyclinD3(h)	100
ALK4(h)	103	CDK7/cyclinH/MAT1(h)	95
ALK6(h)	95	CDK9/cyclin T1(h)	107
Arg(h)	106	ChaK1(h)	120
AMPKα1(h)	104	CHK1(h)	104
AMPKα2(h)	93	CHK2(h)	92
A-Raf(h)	103	CHK2(I157T)(h)	104
ARK5(h)	97	CHK2(R145W)(h)	95
ASK1(h)	112	CK1γ1(h)	103
Aurora-A(h)	107	CK1γ2(h)	97
Aurora-B(h)	106	CK1γ3(h)	109
Aurora-C(h)	100	CK1δ(h)	103
Axl(h)	97	CK2(h)	108
Blk(h)	88	CK2α1(h)	102
Bmx(h)	119	CK2α2(h)	105
BRK(h)	116	CLIK1(h)	108
BrSK1(h)	96	CLK1(h)	106
BrSK2(h)	104	CLK2(h)	103
BTK(h)	90	CLK3(h)	101
BTK(R28H)(h)	98	CLK4(h)	97
B-Raf(h)	110	cKit(h)	98
B-Raf(V599E)(h)	115	cKit(D816V)(h)	102
CaMKI(h)	107	cKit(D816H)(h)	100
CaMKIß(h)	104	cKit(V560G)(h)	94
CaMKIγ(h)	108	cKit(V654A)(h)	92
CaMKIIα(h)	101	CSK(h)	102
CaMKIIβ(h)	104	c-RAF(h)	110
CaMKIIγ(h)	104	cSRC(h)	96
CaMKIδ(h)	94	DAPK1(h)	102
CaMKIIδ(h)	103	DAPK2(h)	111
CaMKIV(h)	93	DCAMKL2(h)	82

Kinase	Activity (% Control)	Kinase	Activity (% Control)
DCAMKL3(h)	101	Flt3(h)	103
DDR1(h)	101	Flt4(h)	106
DDR2(h)	102	Fms(h)	97
DMPK(h)	104	Fms(Y969C)(h)	99
DRAK1(h)	107	Fyn(h)	93
DYRK1A(h)	99	GCK(h)	97
DYRK1B(h)	98	GCN2(h)	114
DYRK2(h)	101	GRK1(h)	100
DYRK3(h)	106	GRK2(h)	104
eEF-2K(h)	108	GRK3(h)	101
EGFR(h)	103	GRK5(h)	107
EGFR(L858R)(h)	102	GRK6(h)	98
EGFR(L861Q)(h)	102	GRK7(h)	100
EGFR(T790M)(h)	100	GSK3α(h)	101
EGFR(T790M,L858R)(h)	99	GSK3β(h)	81
EphA1(h)	92	Haspin(h)	88
EphA2(h)	117	Hck(h)	97
EphA3(h)	104	Hck(h) activated	113
EphA4(h)	96	HIPK1(h)	122
EphA5(h)	108	HIPK2(h)	105
EphA7(h)	84	HIPK3(h)	103
EphA8(h)	123	HIPK4(h)	110
EphB2(h)	83	HPK1(h)	98
EphB1(h)	91	ICK(h)	89
EphB3(h)	90	IGF-1R(h)	92
EphB4(h)	104	IGF-1R(h), activated	107
ErbB2(h)	116	IKKα(h)	107
ErbB4(h)	108	IKKβ(h)	102
FAK(h)	96	IKKε(h)	101
Fer(h)	109	IR(h)	97
Fes(h)	99	IR(h), activated	102
FGFR1(h)	111	IRE1(h)	104
FGFR1(V561M)(h)	101	IRR(h)	103
FGFR2(h)	104	IRAK1(h)	105
FGFR2(N549H)(h)	93	IRAK4(h)	113
FGFR3(h)	97	Itk(h)	117
FGFR4(h)	108	JAK1(h)	100
Fgr(h)	111	JAK2(h)	96
Flt1(h)	99	JAK3(h)	88
Flt3(D835Y)(h)	107	JNK1α1(h)	99

Kinase	Activity (% Control)	Kinase	Activity (% Control)
JNK2α2(h)	94	MRCKβ(h)	103
JNK3(h)	146	MSK1(h)	96
KDR(h)	104	MSK2(h)	101
Lck(h)	119	MSSK1(h)	103
Lck(h) activated	101	MST1(h)	115
LIMK1(h)	103	MST2(h)	100
LKB1(h)	91	MST3(h)	101
LOK(h)	91	MST4(h)	99
Lyn(h)	111	mTOR(h)	93
LRRK2(h)	96	mTOR/FKBP12(h)	108
LTK(h)	107	MuSK(h)	101
MAPK1(h)	111	MYLK2(h)	100
MAPK2(h)	95	MYO3B(h)	103
MAP4K4(h)	101	NEK1(h)	97
MAP4K5(h)	109	NEK2(h)	90
MAPKAP-K2(h)	108	NEK3(h)	100
MAPKAP-K3(h)	103	NEK6(h)	93
MEK1(h)	86	NEK7(h)	94
MEK2(h)	103	NEK9(h)	106
MARK1(h)	97	NIM1(h)	105
MARK4(h)	106	NEK11(h)	94
MEKK2(h)	109	NLK(h)	98
MELK(h)	108	NUAK2(h)	105
Mer(h)	101	p70S6K(h)	98
Met(h)	105	PAK1(h)	107
Met(D1246H)(h)	107	PAK2(h)	101
Met(D1246N)(h)	108	PAK4(h)	102
Met(M1268T)(h)	128	PAK3(h)	91
Met(Y1248C)(h)	109	PAK5(h)	103
Met(Y1248D)(h)	101	PAK6(h)	89
Met(Y1248H)(h)	105	PAR-1Bα(h)	103
MINK(h)	108	PASK(h)	126
MKK6(h)	99	PEK(h)	100
MKK7β(h)	100	PDGFRα(h)	96
MLCK(h)	103	PDGFRα(D842V)(h)	103
MLK1(h)	101	PDGFRα(V561D)(h)	83
MLK2(h)	101	PDGFRβ(h)	103
Mnk2(h)	94	PDHK4(h)	90
MOK(h)	97	PDK1(h)	97
MRCKα(h)	85	PhKγ2(h)	105

Kinase	Activity (% Control)	Kinase	Activity (% Control)
Pim-1(h)	109	Rse(h)	99
Pim-2(h)	116	Rsk1(h)	102
Pim-3(h)	111	Rsk2(h)	91
PKA(h)	110	Rsk3(h)	99
PKAcβ(h)	91	Rsk4(h)	105
PKBα(h)	100	SAPK2a(h)	101
PKBβ(h)	105	SAPK2a(T106M)(h)	122
PKBγ(h)	100	SAPK2b(h)	91
PKCα(h)	100	SAPK3(h)	122
PKCβI(h)	99	SAPK4(h)	112
PKCβII(h)	97	SGK(h)	116
PKCγ(h)	100	SGK2(h)	103
PKCδ(h)	98	SGK3(h)	96
PKCε(h)	100	SIK(h)	88
PKCη(h)	103	SIK2(h)	105
PKCι(h)	101	SIK3(h)	96
PKCμ(h)	100	SLK(h)	97
PKCθ(h)	99	Snk(h)	99
PKCζ(h)	102	SNRK(h)	110
PKD2(h)	95	Src(1-530)(h)	105
PKD3(h)	107	Src(T341M)(h)	111
PKG1α(h)	110	SRPK1(h)	98
PKG1β(h)	93	SRPK2(h)	101
PKR(h)	104	STK25(h)	111
Plk1(h)	100	STK33(h)	104
Plk3(h)	100	Syk(h)	117
PRAK(h)	98	TAK1(h)	96
PRKG2(h)	109	TAO1(h)	100
PRK2(h)	113	TAO2(h)	98
PrKX(h)	101	TAO3(h)	109
PTK5(h)	99	TBK1(h)	109
Pyk2(h)	103	Tec(h) activated	92
Ret(h)	107	TGFBR1(h)	102
Ret (V804L)(h)	107	Tie2 (h)	85
Ret(V804M)(h)	111	Tie2(R849W)(h)	84
RIPK2(h)	106	Tie2(Y897S)(h)	89
ROCK-I(h)	100	TLK1(h)	94
ROCK-II(h)	103	TLK2(h)	96
Ron(h)	89	TNIK(h)	121
Ros(h)	90	TrkA(h)	86

Kinase	Activity (% Control)
TrkB(h)	85
TrkC(h)	112
TSSK1(h)	111
TSSK2(h)	101
TSSK3(h)	108
TSSK4(h)	97
TTBK1(h)	99
TTBK2(h)	100
TTK(h)	94
Txk(h)	99
TYK2(h)	89
ULK1(h)	112
ULK2(h)	108
ULK3(h)	101
Wee1(h)	110
WNK2(h)	105
WNK3(h)	103
VRK2(h)	107
Yes(h)	108
ZAK(h)	100
ZAP-70(h)	109
ZIPK(h)	108
ATM(h)	103
ATR/ATRIP(h)	100
DNA-PK(h)	96
PI3 Kinase (p110b/p85a)(h)	102
PI3 Kinase (p120g)(h)	100
PI3 Kinase (p110d/p85a)(h)	100
PI3 Kinase (p110a/p85a)(h)	101
PI3 Kinase (p110a(E542K)/p85a)(h)	101
PI3 Kinase (p110a(H1047R)/p85a)(h)	101
PI3 Kinase (p110a(E545K)/p85a)(h)	100
PI3 Kinase (p110a/p65a)(h)	101
PI3KC2a(h)	100
PI3KC2g(h)	105
PIP4K2a(h)	102
PIP5K1a(h)	102
PIP5K1g(h)	103
