# Supplementary material for: Nuclear KIT induces a NFKBIB-RELA-KIT autoregulatory loop in imatinib-resistant gastrointestinal stromal tumors
Source: Oncogene. 2019 Jul 30;38(38):6550–65. doi: 10.1038/s41388-019-0900-9 (PMC6756115; doi:10.1038/s41388-019-0900-9)
Supplement: Supplementary file 4 — Supplementary TableS3. [file 41388_2019_900_MOESM4_ESM.pdf]

Table S3. Primers Used in ChIP-seq and ChIP studies

Target	Forward Sequence	Reverse Sequence
KIT	GGCATTAACACGTCGAAAGA	GATCCGAGCTCTGGTCCAC
NFKBIB	ACTGTGTCTGGCCCTGAGTT	CTGGGACTGGAAGAGACAGG
