# Supplementary figures and images for: Nuclear KIT induces a NFKBIB-RELA-KIT autoregulatory loop in imatinib-resistant gastrointestinal stromal tumors
Source: Oncogene. 2019 Jul 30;38(38):6550–65. doi: 10.1038/s41388-019-0900-9 (PMC6756115; doi:10.1038/s41388-019-0900-9)

Figure S1

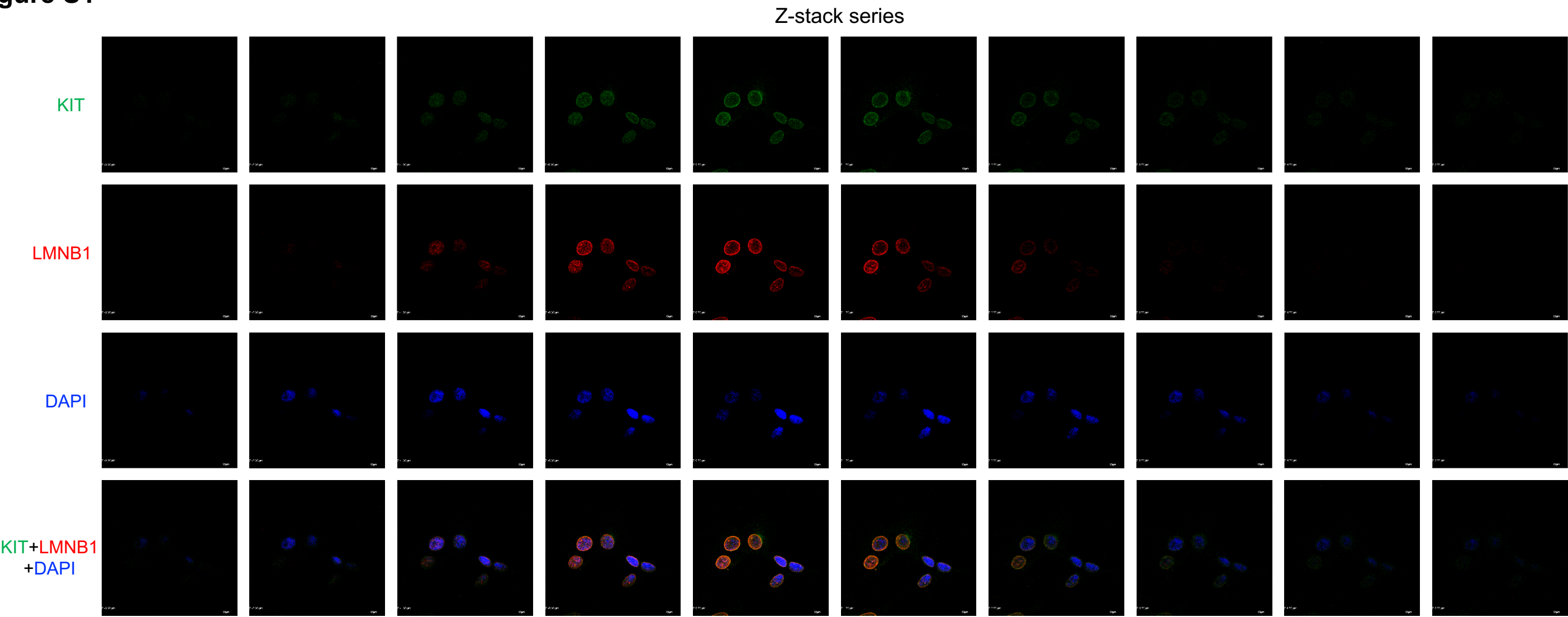

GIST48

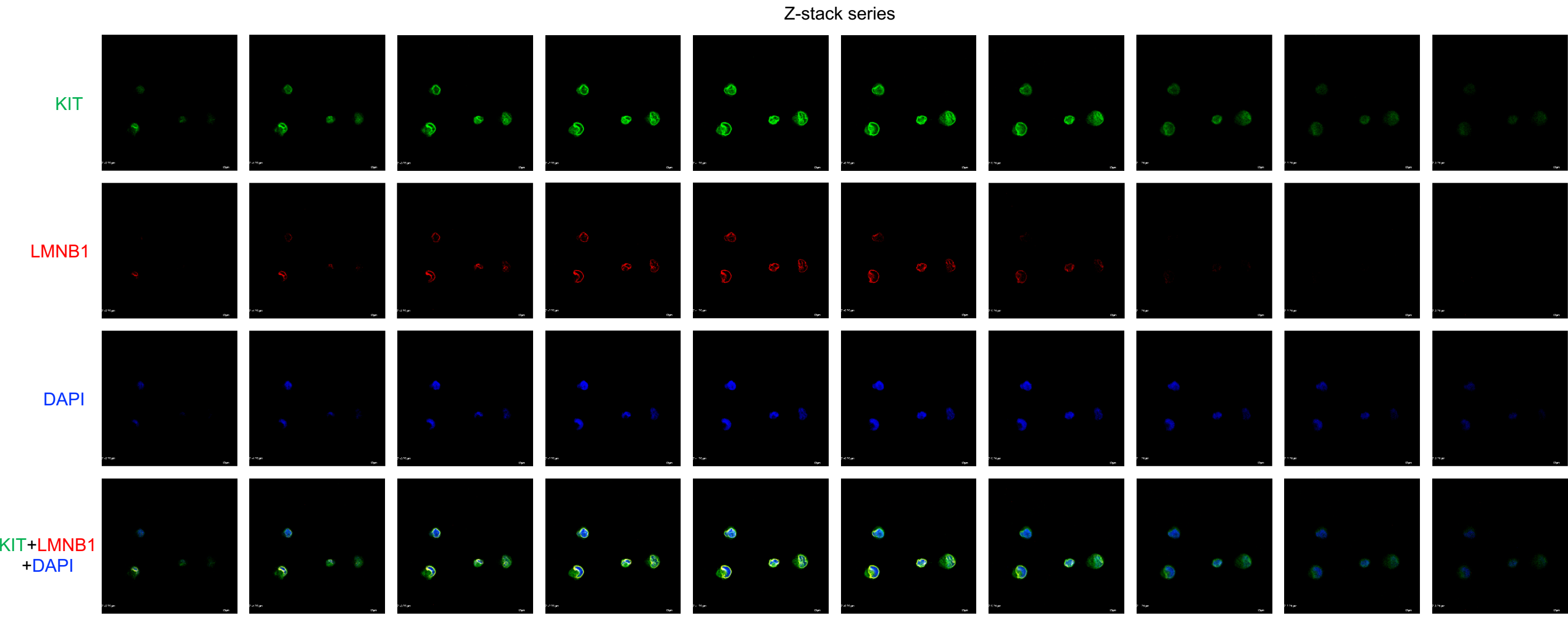

GIST430

Supplement: Supplementary file 6 — Supplementary FigureS1. [file 41388_2019_900_MOESM6_ESM.pdf]

Figure S2

A

Genome distribution of KIT-bound DNA

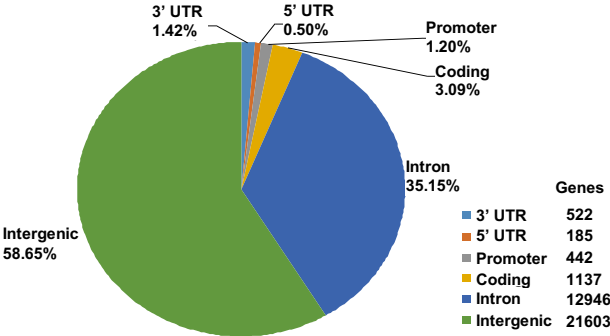

B

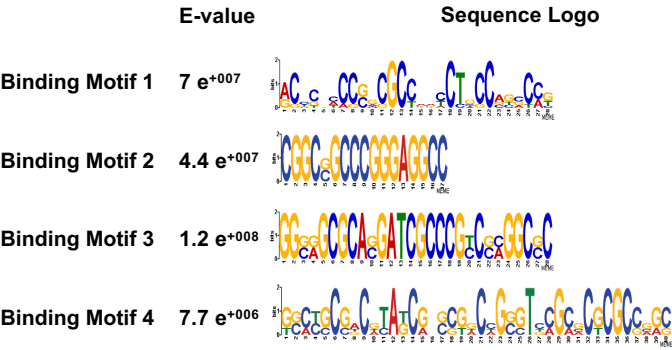

Supplement: Supplementary file 7 — Supplementary FigureS2. [file 41388_2019_900_MOESM7_ESM.pdf]

# Figure S3

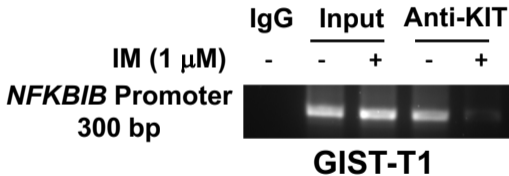

Supplement: Supplementary file 8 — Supplementary FigureS3. [file 41388_2019_900_MOESM8_ESM.pdf]

# Figure S4

**A**

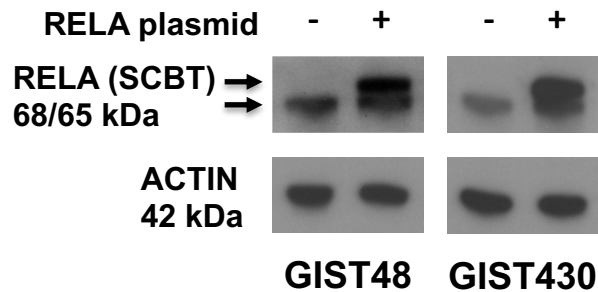

**B**

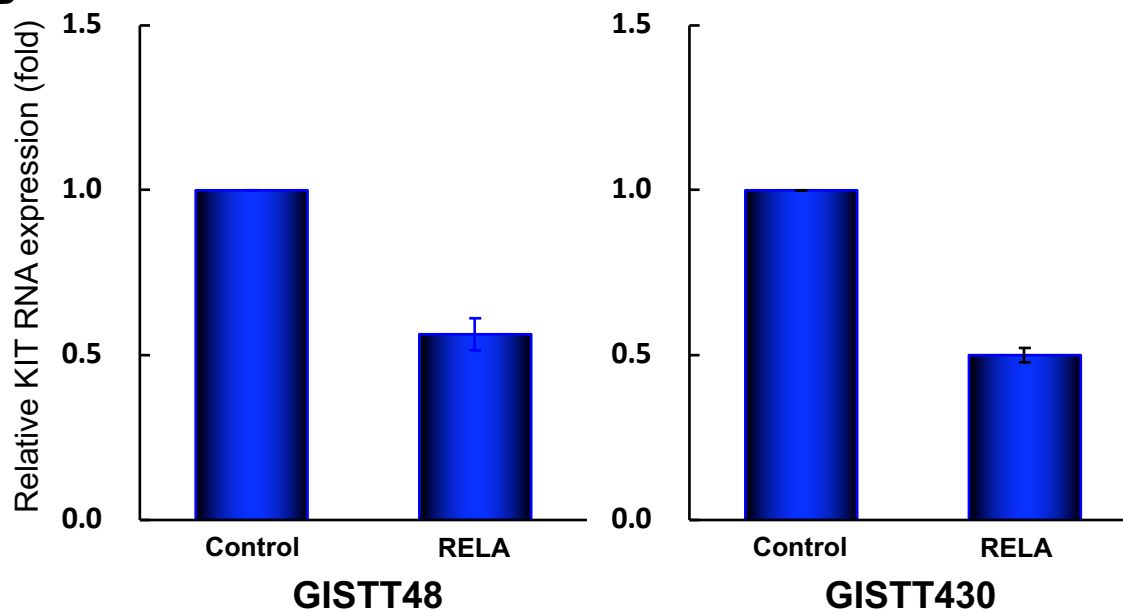

Supplement: Supplementary file 9 — Supplementary FigureS4. [file 41388_2019_900_MOESM9_ESM.pdf]

**Figure S5**

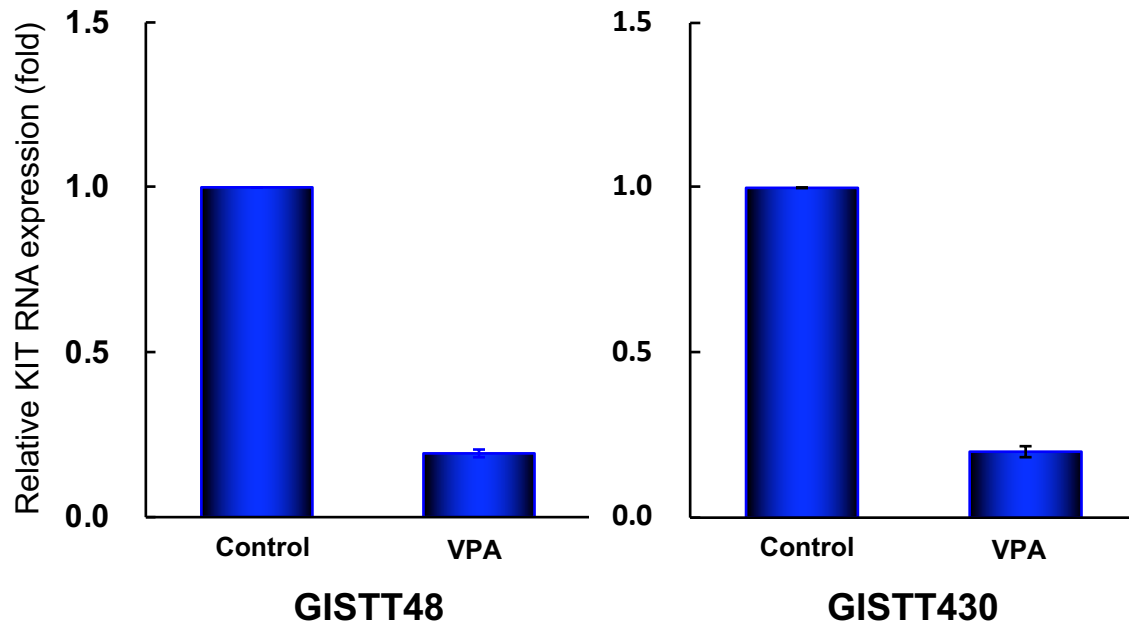

Supplement: Supplementary file 10 — Supplementary FigureS5. [file 41388_2019_900_MOESM10_ESM.pdf]
